# Single‑cell RNA sequencing reveals fibroblast heterogeneity and identifies CLOCK as a key regulator in fibrotic skin diseases

**DOI:** 10.1038/s41598-025-30260-6

**Published:** 2025-12-06

**Authors:** Yue Li, Changmin Li, Wei Liu, Tianbao Gao, Qin Liu, Ling Yang, Songtao Li, Rui Tang, Lei Yang

**Affiliations:** 1 Institute of Burn Research, PLA, State Key Laboratory of Trauma, Burn and Combined Injury, The First Affiliated Hospital of Third Military Medical University (Army Medical University), Chongqing, People’s Republic of China; 2 Analysis Center, College of Basic Medical Sciences, Third Military Medical University (Army Medical University), Chongqing, People’s Republic of China; 3 Osteopaedic department, No.924 Hospital of PLA, Guilin, Guangxi People’s Republic of China; 4 Outpatient department, The First Affiliated Hospital of Third Military Medical University (Army Medical University), Chongqing, People’s Republic of China

**Keywords:** Fibrosis, Fibroblast heterogeneity, Single-cell RNA sequencing, Clock, Migration, Cancer, Computational biology and bioinformatics, Diseases, Genetics, Molecular biology

## Abstract

Fibrosis, characterized by excessive extracellular matrix (ECM) accumulation and fibroblast proliferation, significantly contributes to global morbidity and mortality, affecting millions worldwide. Despite its prevalence, the mechanisms underlying fibrotic skin diseases remain poorly understood, and effective treatments are scarce. This study leverages single-cell RNA sequencing (scRNA-seq) to unravel the heterogeneity of fibroblasts in fibrotic skin diseases, including normal skin, scar, keloid, and scleroderma. Through comprehensive analysis of scRNA-seq data from public repositories, we identified distinct fibroblast subpopulations specific to each fibrotic condition. Notably, pivotal regulators for each sub-fibroblast cluster were discovered: IRF4 for scar, CLOCK for keloid, RUNX3 for scleroderma, and HOXC4 for normal skin. Further, CLOCK was found to be predominantly expressed in keloid tissues, with its upregulation enhancing fibroblast proliferation and migration in vitro. Analysis of The Cancer Genome Atlas (TCGA) data revealed that CLOCK and its regulon genes were upregulated in skin cutaneous melanoma and even more so in metastatic tumors. Our findings underscore the utility of scRNA-seq in dissecting the cellular complexity of fibrotic skin diseases and highlight potential therapeutic targets. This study not only advances our understanding of fibroblast heterogeneity in fibrosis but also opens avenues for targeted therapeutic strategies, moving closer to personalized medicine for fibrotic diseases.

**Supplementary Information:**

The online version contains supplementary material available at 10.1038/s41598-025-30260-6.

## Introduction

Fibrosis, which is characterized by the excessive accumulation of extracellular matrix (ECM) and fibroblast proliferation, represents a major contributor to global morbidity and mortality.^1,2^ Indeed, fibrosis plays a significant role in most cases of organ failure^3^. Examples are wide-ranging: systemic sclerosis (SSc); idiopathic pulmonary fibrosis (IPF); liver cirrhosis; kidney fibrosis; and cardiac fibrosis observed in cardiac hypertrophy resulting in heart failure.^4–8^ Fibrotic skin diseases, such as scleroderma, hypertrophic scar, and keloid, involve the buildup of ECM components in the dermis.^9,10^ The global impact of these diseases is significant, affecting millions of people worldwide. Despite this, the underlying causes of fibrotic skin diseases have not been fully elucidated, and effective treatments are still lacking. In these conditions, fibroblasts play a central role, leading to ECM accumulation, ECM maintenance and reabsorption, wound healing, inflammation, angiogenesis, cancer progression, and in physiological as well as pathological tissue fibrosis^11^. They demonstrate heightened proliferative potential, increased migration and invasion capacity, and elevated ECM deposition, all of which contribute to the pathogenesis of fibrosis.^12–14^ Fibroblasts are mesenchymal cells derived from the embryonic mesoderm tissue, and they are not terminally differentiated^15^. For a long time, it was assumed that fibroblasts were a uniform population of spindle-shaped cells.^11,16,17^ However, emerging evidence indicates that fibroblasts are a morphologically and functionally heterogeneous cell population.

The advent of single-cell RNA-sequencing (scRNA-seq) has provided an opportunity to explore the heterogeneity of fibroblasts in the skin under both homeostatic and pathological conditions^18^. scRNA-seq analyses have suggested that fibroblasts can be subdivided into multiple distinct subgroups in normal human dermis.^19,20^ Furthermore, scRNA-seq has been instrumental in studying the heterogeneity of fibroblasts in various fibrotic diseases, including lung fibrosis, systemic sclerosis, and Dupuytren’s disease.^21–23^ Recent articles have reported that keloid-derived fibroblasts can be categorized into a mesenchymal fibroblast subpopulation and three other subpopulations, with a significantly increased percentage of mesenchymal fibroblasts in keloids compared to normal scars. These mesenchymal fibroblasts are crucial for the excessive expression of collagen in keloids^24^. However, despite the insights gained from studies of fibroblast heterogeneity in other fibrotic diseases, there is a noticeable lack of research utilizing scRNA-seq to investigate the specific heterogeneity of fibroblasts in fibrotic skin diseases.

In this study, we obtained scRNA-seq data of fibrotic skin diseases including normal skin, scar, keloid, and scleroderma. We identified four specific sub-fibroblast populations within each fibrotic disease. Additionally, we identified the pivotal regulators of each specific sub-fibroblast cluster, including IRF4 for scar-related, CLOCK for keloid-related, RUNX3 for scleroderma-related, and HOXC4 for normal skin sub-fibroblast clusters. Further functional studies revealed that CLOCK was mainly expressed in keloid tissues and its upregulation can directly increase the proliferation and migration of fibroblast. Furthermore, analysis of TCGA data on skin cutaneous melanoma revealed that Clock and its regulon genes were predominantly upregulated in tumors compared to adjacent normal tissue. Besides, Clock and its regulon genes were even higher in metastasis tumor compared to tumor. These findings will help us more comprehensively understand fibrotic skin diseases and provide potential different treatment targets for various fibrotic diseases.

## Result

###  Single-cell RNA-seq reveals cell heterogeneity of skin fibrotic disease

**Fig. 1:**
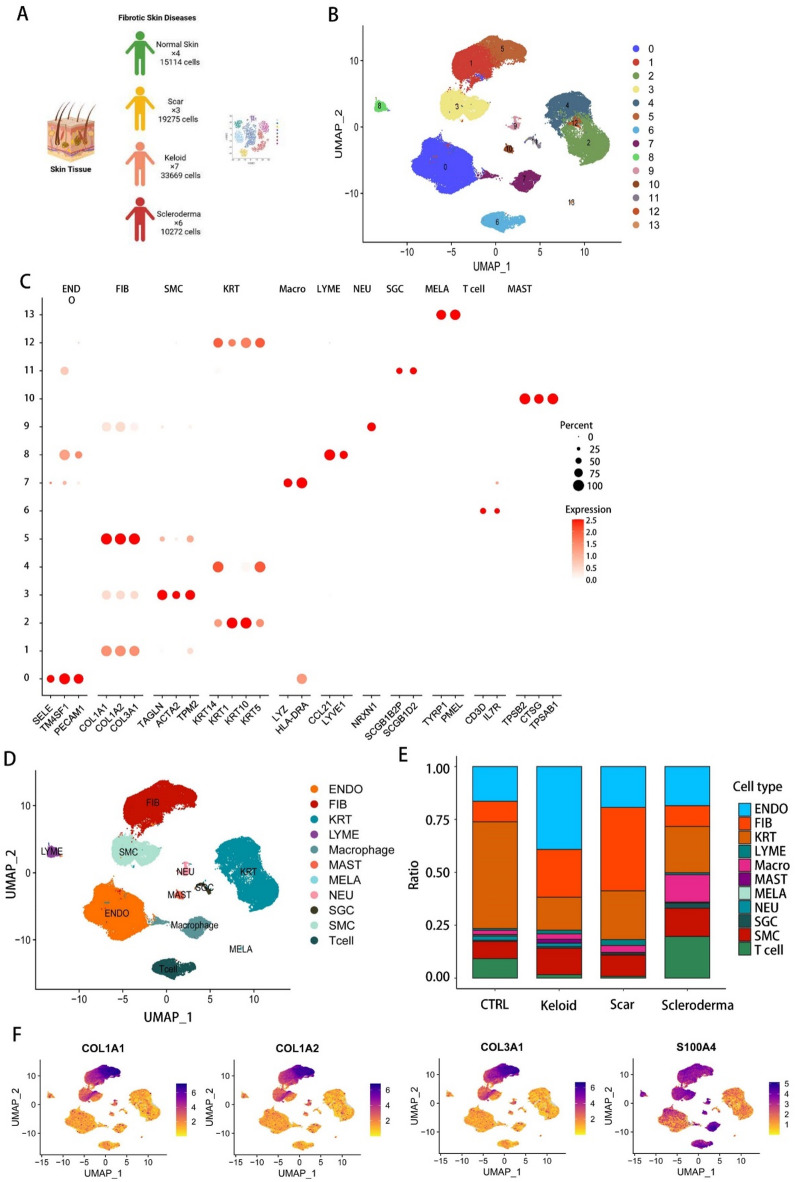
Single-cell RNA-seq reveals cell heterogeneity of skin fibrotic disease. (**A**) Schematic of the experimental workflow for scRNA-seq analysis. (**B**) UMAP embeddings of **78**,**430** cells clustered in 14 groups from 4 skin states. (**C**) Dot plots revealing the expression the 27 gene expression among 14 clusters. The size of the dots represents the proportion of cells expressing the particular marker, and the colour spectrum indicates the mean expression levels of the markers. The specific canonical cluster name was exhibited on the top of plot. (**D**) UMAP embeddings of 11 canonical clusters, endothelial cell (ENDO); fibroblast (FIB); keratinocyte (KRT); lymphatic endothelial cells (LYME); Macrophage; mast cell (MAST); Melanocytes (MELA); neural cell (NEU); sweat gland cells (SGC); smooth muscle cells (SMC); T cell (T cell). (**E**) Histogram depicting the relative proportion of each cell cluster among the respective sample states in all lesions as indicated. (**F**) Feature plots of expression distribution for selected cluster-specific genes. Expression levels for each cell are color-coded and overlaid onto the plot.

To gain a better understanding of human skin fibrotic disease, we took advantage of a public single-cell database and collected data on 20 human skin fibrotic-related samples, including normal skin tissue (CTRL, *n* = 4), skin scar tissue (SC, *n* = 3), skin keloid tissue (KL, *n* = 7) and skin scleroderma tissue (SCLE, *n* = 6) (Fig. 1A). After quality control and rigorous filtration, we obtained the transcriptomes of 78,330 cells (CTRL:15,114; SC:19,275; KL:33669; SCLE:10272) (Fig. 1A and SM. Figure 1A). Unsupervised Uniform Manifold Approximation and Projection (UMAP)-clustering revealed 14 main cell clusters (Fig. 1B), which were classified as transcriptional cluster proximity via a phylogenetic cluster tree (SM. Figure 1B). The individual clusters were defined by comparison to known lineage or canonical markers. As a result, the 14 clusters could be divided into 11 canonical cell types including endothelial cell with high expression of SELE, TM4SF1 and PECAM1^25^.The fibroblast lineage characterized by high COL1A1, COL1A2 and COL3A1 (Fig. 1C and F). The smooth muscle cell specifically expressing TAGLN, ACTA2 and TPM2, keratinocyte characterized by high KRT1, KRT5, KRT10 and KRT14, macrophage with high expression of LYZ and HLA-DRA, lymphatic endothelial cell specifically expressing CCL21 and LYVE1, neural cell characterized by high NRXN1, sweat gland cells with high expression of SCGB1D2 and SCGB1B2P, melanocyte with high expression of TYRP1 and PMEL, T cell with high expression of CD3D and IL7R, and mast cell specifically expressing TPSAB1, TPSAB2 and CTSG (Fig. 1C and D and SM. Figure 1C)^25^. We next aimed to determine the compositional differences in cell types among the four sample types. Based on the findings from dimensionality reduction clustering, it was observed that the cell types in all four fibrosis-related samples were consistent (Fig. 1E). Additionally, the analysis revealed that normal skin tissue predominantly consisted of keratinocytes, whereas keloid samples were mainly composed of endothelial cells, and scar samples were predominantly composed of fibroblasts (Fig. 1D, and 1F). These results indicated that keloids may have a richer blood vessel or blood supply, while fibroblasts play a crucial role in the healing process of skin injuries. Therefore, fibroblasts are the primary focus of our investigation, as we aim to uncover potential insights into the development of fibrotic diseases at the single cell level. The proportion of fibroblast exhibited dramatic differences among several conditions. Suggesting that development of fibroblast undergo significant change during different environment.

### Identification of diseases specific fibroblast among four skin fibrotic states

**Fig. 2:**
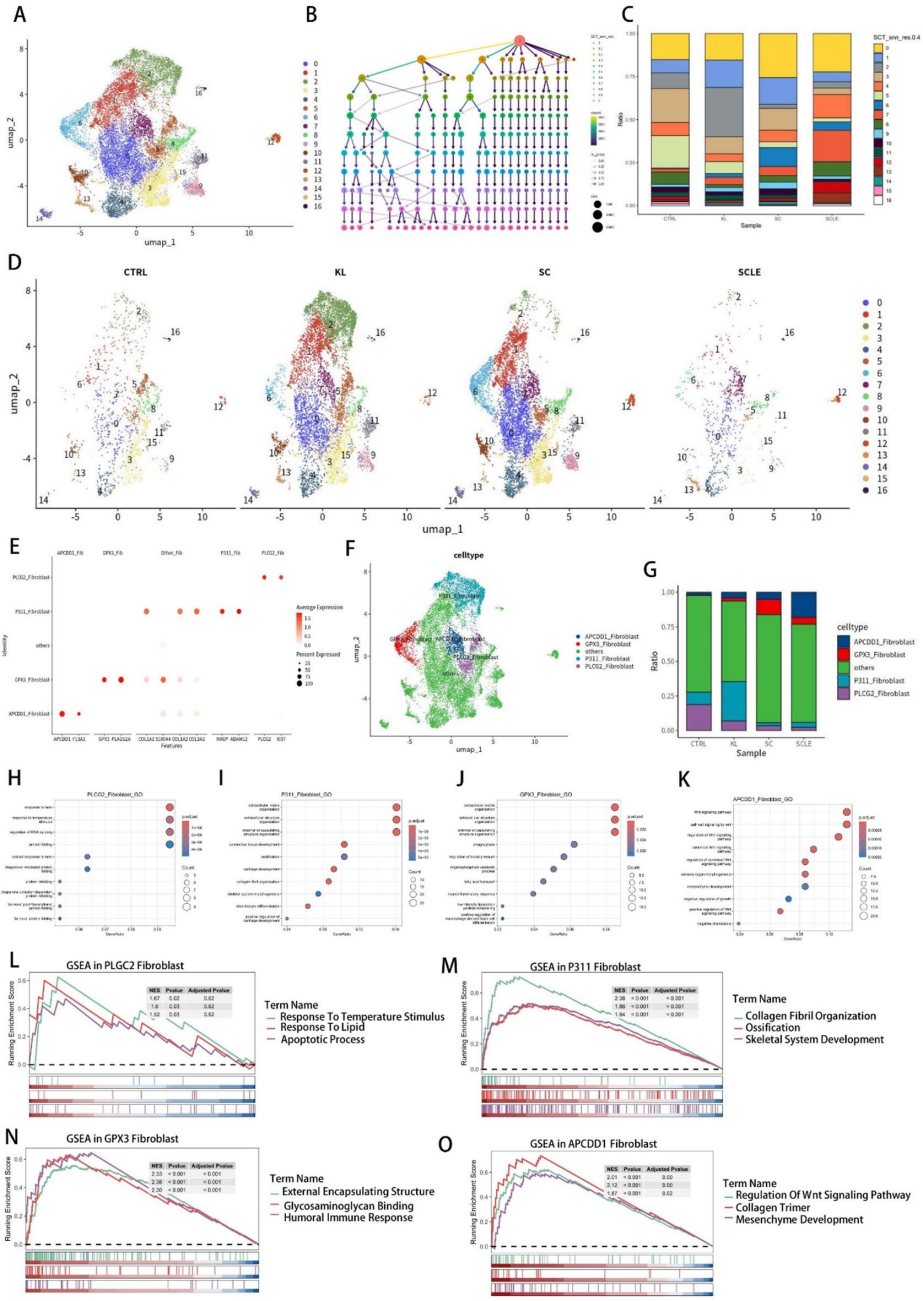
Identification of diseases specific fibroblast among four skin fibrotic states. (**A**) UMAP of 17,647 reclustered fibroblasts from all sapiens; (**B**) Clustering tree plot for the reclustered fibroblasts using various resolution parameters (from 0.1 to 1); (**C**) The proportion of each reclustered fibroblast among normal skin (CTRL), keloid (KL), scar (SC) and scleroderma (SCLE) (resolution = 0.4); (**D**) The UMAP plot divided by 4 distinct sample states (CTRL, KL, SC, and SCLE) depicting all identified fibroblasts; (**E**) Dot plots showing the expression of the specific fibroblast markers genes across 5 subclusters (The size of the dots represents the proportion of cells expressing the particular marker, and the color spectrum indicates the average expression levels of the markers); (F) UMAP visualization of the clustered fibroblast scRNA-seq profile, including APCDD1_ fibroblast, GPX3_ fibroblast, other fibroblast, P311_fibroblast, and PLCG2_ fibroblast; (**G**) The proportion of five reclustered specific fibroblast among 4 different sample states; (**H**,** I**,** J and K**) GO biological process enrichment results of four specific fibroblast clusters DEGs; The x-axis represents the gene ratio, and the y-axis represents the GO terms (PLCG2_ fibroblast, P311_fibroblast, GPX3_ fibroblast and APCDD1_ fibroblast); (**L**) GSEA analysis of PLGC2 Fibroblast in GO terms including Response to temperature stimulus, response to lipid and apoptotic process; (**M**) GSEA analysis of **P311** Fibroblast in GO terms including collagen fibril organization, ossification and skeletal system development; (**N**) GSEA analysis of GPX3 Fibroblast in GO terms including external encapsulating structure, glycosaminoglycan binding, and humoral immune response; (**O**) GSEA analysis of Apcdd1 fibroblast in GO terms including regulation of Wnt signaling pathway, collagen trimer and mesenchyme development.

Given that fibroblasts are the primary focus of our investigation, we aim to uncover potential insights into the development of fibrotic diseases at the single-cell level. After defining clusters, we isolated 17,647 high-quality fibroblasts from all samples (Fig. 2A). To analyze the heterogeneity and characteristics of fibroblasts in detail, we further segregated the cells into 17 subclusters using a phylogenetic cluster tree (Fig. 2B). Based on the proportion of each sub-fibroblast group across four disease states, we identified four specific disease-related subclusters: cluster 5 in CTRL, cluster 2 in KL, cluster 6 in SC, and cluster 7 in SCLE (Fig. 2B, C and D). These subclusters were annotated based on the calculation of differentially expressed genes (DEGs) using canonical markers which were specifically high expressed in those subclusters, such as *PLCG2*, *P311*, *GPX3*, and *APCDD1* (Fig. 2E and F). In this study, fibroblasts were categorized into five groups: four disease-specific such as PLCG2_Fibroblast, P311_Fibroblast, GPX3_Fibroblast, and APCDD1_Fibroblast and one representing other fibroblasts (Fig. 2G). To elucidate the roles of each disease-specific fibroblast group further, Gene Ontology (GO) and Gene Set Enrichment Analysis (GSEA) were performed on the differentially expressed genes of these cell groups (Fig. 2H and O). The observed differences proved to be both highly significant and intriguing. For PLCG2_Fibroblast, the GO results revealed that it mainly enriched in response to heat, response to temperature stimulus, and protein folding (Fig. 2H). This indicates that upon exposure to thermal stimulation in unfavorable environments, these cells can rapidly initiate the production of HSP-related proteins. Furthermore, GSEA pathway enrichment analysis revealed that the PLCG2_Fibroblast cell group not only responds strongly to temperature stimulation but also shows enrichment in the pathways associated with cell apoptosis and lipid metabolism response (Fig. 2L). The analysis of P311_Fibroblast revealed enrichment in pathways related to extracellular matrix reconstruction, ossification, and cartilage development (Fig. 2I). Additionally, the GSEA analysis indicated that P311_Fibroblast exhibited enrichment in collagen fibril organization, ossification, and skeletal system development (Fig. 2M). These findings indicate that P311_Fibroblast has a propensity to differentiate into osteoblasts and exhibit a more mesenchymal phenotype, which is highly relevant to the clinical manifestation of keloid sclerosis (Fig. 2I and M). GO and GSEA analyses also suggested that extracellular matrix organization, glycosaminoglycan binding, fatty acid transport, and humoral immune response were enriched in GPX3_fibroblast (Fig. 2J and N). The scleroderma specific APCDD1_fibroblasts were mainly enriched in Wnt signal pathway, mesenchyme development, and collagen trimer via GO and GSEA analysis (Fig. 2K). These results suggest that APCDD1_fibroblasts may be closely related to osteogenesis and mesenchyme development, which could explain the clinical characteristics of scleroderma, including the ectopic fibrotic phenotype and mesenchyme transformation (Fig. 2O). Above all, by integrating and comparing the differences in transcript levels of fibroblasts across various fibrotic diseases, we identified distinct fibroblast populations associated with each disease and conducted initial investigations into their cellular characteristics. Most of the results from the enrichment analysis align with the clinical manifestations of the respective diseases, providing further evidence for the significance of fibroblasts in skin fibrosis.

###  Interaction networks of fibroblasts among individual microenvironments

**Fig. 3:**
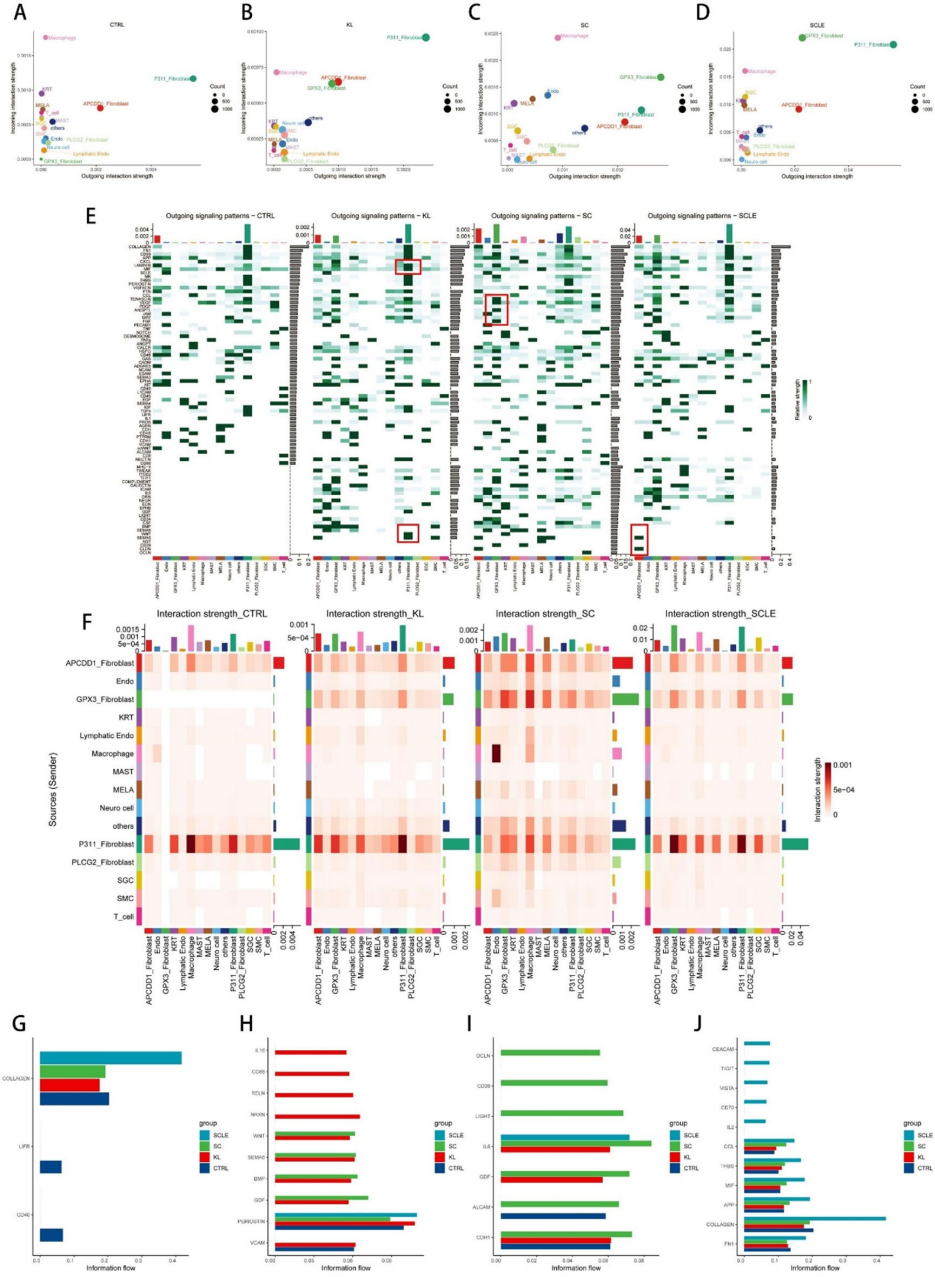
Communication analysis among individual fibrotic microenvironments. (**A**,** B**,** C and D**) Compared the outgoing and incoming interaction strength in 2D space to identify the cell populations with significant changes in sending or receiving signals among CTRL, KL, SC, and SCLE (Count refers to the number of inferred receptor-ligand pairs associated with each cell group. Cell types with high outgoing interaction strength are expected to be initiators of cell–cell interactions, and cell types with high incoming interaction strength are expected to be targets of cell–cell interactions); (**E**) Outgoing patterns for signaling of all cell types in each group including CTRL, KL, SC, and SCLE; (**F**) Differential number of interaction strength, visualized as a heatmap. The top-colored bar plot represents the sum of a column of values displayed (incoming signaling). The right colored bar plot represents the sum of a row of values (outgoing signaling). (**G**,** H**,** I**,** and J**) Relative information flow from cell–cell interaction analysis. The specific receptor-ligand pathways were enriched in CTRL (blue bar), KL (red bar), SC (green bar), and SCLE (sky blue bar) sapiens.

In order to explore the fibroblast cell interaction network in the specific environments of individual diseases, we utilized CellChat, a cell ligand/receptor pairing-based database, to evaluate the strength of cell-cell communication and the level of each signaling pathway^26^. Firstly, we analyzed the outgoing and incoming interaction capabilities of all fibrotic diseases and found that the P311_fibroblast served as the primary source of the output signal in normal skin tissues (Fig. 3A). In keloid tissue, P311_fibroblast plays a crucial role in both signal reception and transmission (Fig. 3B). GPX3_fibroblast emerged as the strongest signal sender, with macrophages functioning as the main signal receivers in scar tissue (Fig. 3C). In scleroderma, GPX3_fibroblasts play a vital role as signal receivers, while P311_fibroblasts function as the main signal source (Fig. 3D). In keloid samples, the LAMININ, MIF, SEMA6, and WNT cell interaction signaling pathways were specifically identified as being secreted by P311_fibroblast (Fig. 3E). In scleroderma samples, CLDN and WNT signals were specifically identified as being secreted by APCDD1_fibroblasts (Fig. 3E). Subsequently, we investigated the potential interacting cell types; in addition to fibroblasts, macrophages were identified as crucial signaling targets (Fig. 3F). We conducted a thorough comparison and analysis of the pathways associated with the four disease states (Fig. 3G and J). These results indicate that the keloid-specific fibroblast cluster, P311_fibroblast, acts as the main source and key factor in fibrotic diseases.

###  Identification the key transcription regulator of individual specific sub-fibroblasts

**Fig. 4:**
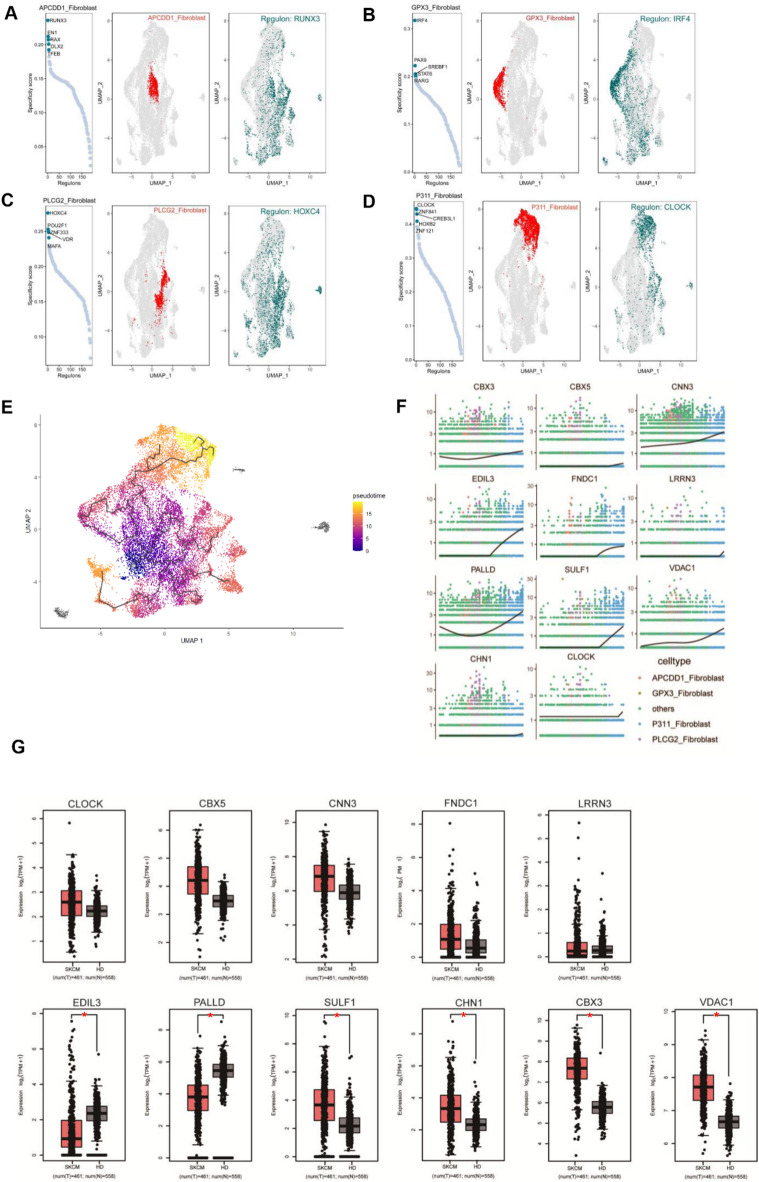
Identification the key transcription regulator of individual specific sub-fibroblasts. (**A**,** B**,** C**,** and D**) Cell-type-specific regulon activity analysis. L**eft plot**: Rank for regulons in PLCGG2_fibroblast (**A**), GPX3_ fibroblast (**B**), APCDD1_ fibroblast (**C**), P311_fibroblast (**D**) based on regulon specificity score (RSS); **Middle plot**: fibroblast cells are highlighted in the UMAP map (red dots); **Right plot**: Binarized regulon activity scores (RAS) (do Z score normalization across all samples, and set 2.5 as cutoff to convert to 0 and 1) for top regulon Lmo2 on t-SNE map (dark green dots). (**E**) UMAP visualizing the pseudotime trajectory of the aggregated dataset for fibroblast cells; (**F**) Spline plots representing expression of Clock and its regulons with large transitions along the pseudotime. All plots were generated using Monocle3 0.1.2. (**G**) GEPIA expression analysis of Clock and its regulons (11 genes) in human skin cutaneous melanoma patients. Orange box: 461 skin cutaneous melanoma patients (SKCM); blue box: 558 healthy donors (HD). *, *P* < 0.05; **, *P* < 0.01.

To further investigate the regulators dominating cell fate of these four sub-fibroblasts, we applied SCENIC to uncover the gene-regulatory networks and key transcription factors in each fibroblast. In the fibroblasts specific to scleroderma skin tissue, we constructed a gene expression network and calculated the scores of all regulons in this group. The results indicated that RUNX3, EN1, RAX, and DLX2 had higher specificity scores (Fig. 4A). Based on the expression of each transcription factor, RUNX3 was identified as the key regulator of the scleroderma-specific APCDD1_fibroblast (Fig. 4A). Similarly, we identified HOXC4 as a key regulator of PLCG2_fibroblast; however, the expression of HOXC4 was not specific and was found in a certain proportion of most fibroblasts (Fig. 4C). We also determined IRF4 as a fate-determining regulator of scar tissue GPX3_Fibroblast (Fig. 4B). Interestingly, we observed that the specificity score of CLOCK in P311_fibroblast was particularly high, with its expression showing a significant tendency towards APCDD1_fibroblast (Fig. 4D). To further investigate the potential relationships between the different cell types, we conducted a pseudotime trajectory analysis via Monocle3. The pseudotime trajectory revealed that P311_fibroblast was located at the ending stage of the entire differentiation algorithm (Fig. 4E). We observed a relatively high level of CLOCK expression at the end of the pseudotime, in accordance with the differentiation site of P311_fibroblast (Fig. 4F). Notably, CLOCK expression is significantly elevated in the P311_Fibroblast group compared to the others, suggesting its potential importance in fibroblast differentiation (SM. Figure 2). Furthermore, we detected the trend of transcription changes in its regulated genes via pseudotime trajectory. Strikingly, the expression pattern of *CBX3*,* CBX5*,* CNN3*,* EDIL3*,* FNDC1*,* LRRN3*,* PALLD*,* SULF1*,* VDAC1*, and *CHN1* was concordant with that of *CLOCK*, suggesting that these genes may be regulated by *CLOCK* and play critical roles in P311_fibroblast differentiation and function (Fig. 4F). Traditionally, keloids have been characterized as chronic progressive dermal pseudo-tumors that can grow considerably in volume and surface area but do not invade other tissues^27^. To investigate the eleven key keloid genes in skin cancer, we performed a comparative analysis using RNA-seq data from the TCGA database (Fig. 4G). Our analysis revealed that CLOCK exhibited higher expression in SKCM compared to healthy donor samples, but this difference was not statistically significant. Similar results were obtained for CBX5, CNN3 and FNDC1, while LRRN3 displayed no difference in expression at all. In contrast, CBX3, CHN1, SULF1 and VDAC1 were significantly elevated in SKCM compared to HD samples, while EDIL3 and PALLD demonstrated significantly lower expression levels in the cancer samples. Above all, our data indicated that CLOCK might play critical roles in the development and progression of keloids and skin fibrotic tumors.

###  Decreased CLOCK inhibited fibroblast proliferation and migration

**Fig. 5:**
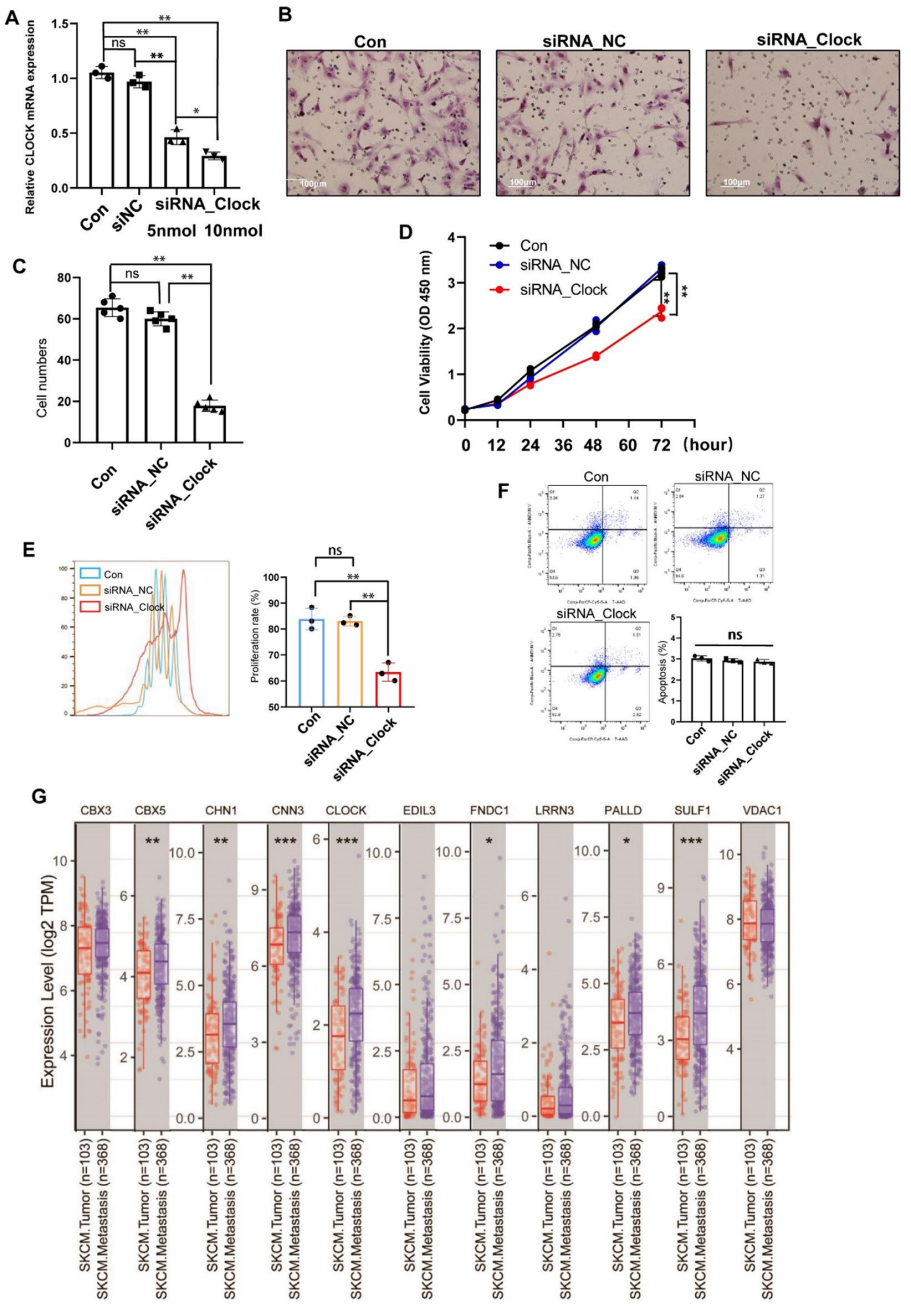
Decreased CLOCK inhibited fibroblast proliferation and migration. (**A**) The relative mRNA expression of clock with clock siRNA (siRNA_clock with 5 nM, and siRNA_clock with 10 nM) and control siRNA; (**B**)Transwell assay showing the migration ability of HSF in 3 group including control, siNC, and siRNA_clock with 10 nM; (**C**) Quantification and comparison of HSF number after transwell, 24 h; (**D**) Cell viability of HSF with siRNA clock transfection was evaluated by CCK-8 assay; Data are shown as the mean ± SD. *N* = 3. (**E**) Flow cytometric analyses of CFSE fluorescence in HSF cell line with siRNA clock transfection at day 5 days after CFSE labeling. (**F**) Flow cytometric analyses of siRNA clock transfection on apoptosis of HSF cells. (**G**) Relative gene expression analysis of Clock and its regulons (11 genes) in human skin cutaneous melanoma patients (SKCM Tumor) and SKCM metastasis patients (SKCM Metastaisis) (TIMER databases). Red box: 103 skin cutaneous melanoma patients (SKCM); purple box: 368 metastasis patients. *, *P* < 0.05; **, *P* < 0.01.

To elucidate the biological function of the *CLOCK* gene in fibroblasts, we utilized siRNA technology to create a specific interference sequence targeting the *clock* gene, which was then transfected into fibroblast cells (Fig. 5A). Subsequent analysis using a transwell assay revealed a marked decrease in cell migration capabilities within the siRNA_CLOCK treated group (Fig. 5B and C). Moreover, a proliferation assay employing CCK8 indicated a significant reduction in cell proliferation rates in the siRNA_CLOCK group after three days of incubation (Fig. 5D). This observation was further supported by results from the CFSE assay, which demonstrated a notable decrease in the number of proliferative generations in the group subjected to CLOCK gene interference (Fig. 5E). Additionally, we assessed fibroblast cell apoptosis rates following CLOCK gene downregulation using Annexin V/propidium iodide staining, revealing no significant difference in the percentage of early apoptotic cells across the three groups (Fig. 5F). To assess the expression of four putative CLOCK targets (EDIL3, PALLD, SULF1, and VDAC1) in the siCLOCK-treated cells, RT-qPCR were performed. The results exhibited that only SULF1 exhibited a significant downregulation in expression within the siCLOCK-treated cells. The expression levels of EDIL3, PALLD, and VDAC1 did not show any notable changes under the same conditions (SM. Figure 3).

Expanding our investigation to the expression of CLOCK and its ten key regulated genes in skin cancer and metastatic samples, we utilized TIMER2.0 for analysis. The results showed that the expression levels of CLOCK, along with CBX5, CHN1, CNN3, FNDC1, PALLD, and SULF1, were significantly elevated in metastatic cancer tissues compared to primary cancer tissues (Fig. 5G). These findings underscore the pivotal role of CLOCK and its associated genes in the progression of skin fibrosis, tumor development, metastasis, and cell migration. Importantly, this research highlights the potential of targeting CLOCK for early intervention in the treatment of excessive skin fibrosis, offering a novel avenue for clinical management.

## Discussion

Despite thorough research into skin fibrosis, the primary mechanisms responsible for their onset remain elusive. Furthermore, options for the prevention and management of skin fibrosis are limited and largely ineffective. The tissue affected by skin fibrosis is characterized by a variety of cell subpopulations, each possessing unique genetic and phenotypic traits. The origins of this heterogeneity in the development of fibrosis remain to be elucidated.

Skin fibrosis is the deposition of excessive extracellular matrix and can occur as part of the dermal injury following burn, surgery, trauma, infection, or radiation, or as a consequence of diseases such as scleroderma and graft-versus-host disease.^28–30^ When skin fibrosis becomes excessive, hypertrophic scars or keloids form. Fibroblasts, crucial in all wound healing stages, are the predominant mesenchymal cells in the dermis of the skin^31^. Traditionally viewed as a uniform and stationary population of spindle-shaped cells, recent evidence reveals their morphological and functional diversity^32^. This shift in perspective has highlighted the significant impact of fibroblast heterogeneity on skin homeostasis and pathological conditions like scarring and fibrosis^33^.

In this study, we utilized single-cell sequencing data from skin fibrosis of varying degrees and types to analyze and compare fibroblasts, identifying their genetic characteristics and fibroblast subpopulations specific to various diseases. Following this, we employed Cellchat analysis to delineate the interactions and key receptor-ligand pairs between disease-specific fibroblasts and other cell groups. Subsequently, SCENIC was applied to identify key regulatory factors of subpopulation-specific fibroblasts. Among these, CLOCK was identified as a critical regulatory factor in fibroblasts within keloid tissues, and further analysis of the TCGA database revealed elevated expression of CLOCK and its downstream genes in skin tumors. Additional in vitro experiments demonstrated that knockdown of CLOCK inhibited the proliferation and migration capabilities of fibroblasts without affecting apoptosis levels (Fig. 5A, B, C, D, E and F). Further database analyses indicated an increase in the expression levels of CLOCK and its downstream genes in metastatic skin cancers (Fig. 5G). Since advanced experimental methodologies have revealed substantial diversity and functional variances amongst fibroblasts during fibrotic processes in organs.^34,35^ Our study utilized cutting-edge single-cell sequencing technology to uncover the diversity of fibroblasts across various skin fibrosis tissues, identifying distinct tissue-specific fibroblast populations. The findings from this segment of our research offer a preliminary insight into the cellular and genetic landscape of skin fibrosis, revealing crucial transcription factors and genes within specific fibroblast subgroups. These results lay the foundation for future investigations into fibrosis progression and alterations in fibroblast functionality, while also pinpointing potential molecular targets for pharmacological interventions.

In this study, we identified *clock* as a pivotal regulator in keloid specific fibroblasts, and function in fibroblast migration and proliferation. The circadian clock is a molecular mechanism for time-keeping that is evolutionarily conserved, regulating daily oscillations in biological processes and behaviors^36^. As *clock* is vital to maintaining physiologic homeostasis and normal function of all organisms. The World Health Organization identified circadian disruption as a probable carcinogen.^37,38^ Articles have reported that ectopic clock expression closely related to cell proliferation and migration in colorectal cancer and breast cancer.^39,40^ These reporting is consistent to our conclusion.

Previously, scholars assumed that the location of fibroblasts influenced gene expression and heterogeneity, but this approach did not allow for a direct comparison of differences between fibroblasts^41^. With the advent of high-throughput sequencing at the single-cell level in tissues, a more detailed subpopulation analysis and comparison enable us to differentiate the heterogeneity among fibroblasts from the dimensions of transcription and transcription factors. This approach is closer to the biological essence and facilitates the monitoring of the biological behavior, morphology, and functional changes of fibroblasts.^42,43^ In this study, we leveraged publicly available scRNA-seq datasets to overcome limitations in sample accessibility and cost, enabling a comprehensive cross-disease analysis of normal skin, scar, keloid, and scleroderma tissues. This methodology not only complements bulk RNA-seq by revealing cellular heterogeneity but also allows for the identification of disease-specific subpopulations and regulators (e.g., CLOCK in keloid fibroblasts) without the need for de novo sample collection, thereby accelerating insights into fibrotic mechanisms while acknowledging potential biases in public data such as variability in sample diversity. Fibroblast diversity shows promise in disease diagnosis and severity assessment. In a study of 61 scleroderma patients, skin biopsies revealed significant expression heterogeneity, aiding in disease severity stratification and treatment response prediction^44^. Understanding the specific roles of fibroblast subpopulations in fibrosis will optimize treatment regimens, leading to improved targeted therapies.

Our research results have several key shortcomings, such as the stability and diversity of sample size, lack of functional validation, longitudinal data, clinical relevance, and technical limitations. The utilization of scRNA-seq data from public repositories inherently limits control over sample size, diversity, and quality. The unspecified diversity in ethnicity, age, and gender among the samples might constrain the generalizability of our findings. Expanding the sample size and ensuring its diversity are essential steps towards validating the identified fibroblast subpopulations across different populations, thereby enhancing the robustness and applicability of our results. What’s more, our study identifies crucial regulators within specific sub-fibroblast clusters based on gene expression profiles, yet it lacks direct functional validation of these regulators in the context of fibrotic skin diseases. Incorporating experimental validation techniques, such as gene knockdown or overexpression studies in cell lines or animal models, is imperative to solidify the causal roles of these regulators in fibrosis, thereby strengthening the foundation of our findings. Fibrosis, being a condition that can impact various organs and tissues, necessitates a broader understanding of fibroblast heterogeneity and regulatory mechanisms across different fibrosis types. Such comparisons could unveil deeper insights into the universal and unique aspects of fibrotic diseases, enriching our comprehension of fibrosis. Although potential targets for therapeutic intervention have been identified, our study falls short of providing direct evidence of clinical efficacy or correlation with disease severity and patient outcomes. Future research endeavors should focus on correlating the presence or activity of specific fibroblast subpopulations with clinical parameters and assessing the therapeutic efficacy of targeting these identified regulators in clinical settings. Such studies would bridge the gap between bench research and bedside application, moving closer to personalized medicine. And the power of scRNA-seq comes with its set of limitations, including sensitivity to technical variability and the challenge of capturing transient or lowly expressed genes. Employing CellChat for analyzing cell-cell communication provides insights based on known ligand-receptor interactions, yet it may overlook novel or context-specific interactions. Employing CellChat for analyzing cell-cell communication provides insights based on known ligand-receptor interactions, yet it may overlook novel or context-specific interactions.

Fibroblasts exhibit heterogeneity and have been extensively studied in murine skin, highlighting the need for further investigation in human skin. Recent studies have identified multiple subsets of fibroblasts. In this study, we conducted preliminary exploration of these specific populations and their distinct roles in fibrosis. Deciphering the regulatory signals of distinct fibroblast subpopulations will facilitate the development of novel therapies for scar prevention, fibrosis treatment, and wound healing enhancement. Hence, future research on unraveling the heterogeneity of human fibroblast subpopulations holds promise for advancing fibroblast cellular therapy in regenerative medicine.

## Methods

### scRNA-seq data download and preprocessing

Single-cell transcriptomics datasets comprising four fibrotic skin states including normal skin, scar, keloid and scleroderma were collected from public repositories. Human skin tissue single-cell sequencing data were obtained from the GEO database and GSA (GSE163973 contains 3 keloids sapiens and 3 scar sapiens, PRJCA003143 including 4 normal skin sapiens and 4 keloid sapiens, GSE160536 contains 6 scleroderma sapiens). The data matrix of all sapiens in PRJCA003143 were directly emailed by Dr. Liu from Peking Union Medical College Hospital.

### Data dimensionality reduction and clustering

The Seurat R package (version 4.2) was utilized to process raw gene expression matrices in the following manner. Cells were filtered based on the following criteria: (1) fewer than 200 unique molecular identifiers (UMIs), over 6,000 or less than 500 expressed genes, or over 20% UMIs derived from the mitochondrial genome; (2) an average expression level of less than 2 for a curated list of housekeeping genes. The gene expression matrices of the remaining high-quality cells were then normalized to the total cellular UMI counts and scaled (scale.factor = 1e4) by regressing out the total cellular UMI counts and percentage of mitochondrial genes. Highly variable genes were identified using the Seurat FindVariableGenes function with default parameters except for “x.low.cutoff”=0.0125 and y.cutoff = 0.5. Subsequently, PCA was performed using the highly variable genes, and significant PCs (top 50) were selected for dimension reduction. Clusters were identified using the FindClusters function (dims.use = 1:40, resolution = 0.2). Finally, tSNE and UMAP analysis was employed for dimension reduction and visualization of gene expression [14], in accordance with the standards of scientific publications to avoid redundancy. Cell cluster were identified according to the article reported by Yang et al.

After extracting all the fibroblasts, we conducted dimensionality reduction and cell clustering once more. To group the fibroblasts, we utilized the “Clustree” package and set the Resolution value to 0.4, resulting in a total of 17 fibroblast groups. Subsequently, we selected specific fibroblasts based on the proportion of each group in the four disease types. To identify markers, we employed the “Findallmarkers” package to identify the specific genes associated with these cell groups.

### Enrichment analysis

Functional enrichment analysis was conducted using Gene Ontology (GO), and single-gene Gene Set Enrichment Analysis (GSEA) with a significance threshold of p-value < 0.05. The R package “clusterProfiler” ans “GseaVis” was utilized for these analyses, in line with the conventions of scientific publications to avoid duplication of information.

Cell–cell communication analysis.

### Trajectory analysis

Pseudotime trajectory of fibroblast was carried out using the workflow suggested in the Monocle3 tutorial (http://cole-trapnell-lab.github.io/monocle-release/monocle3/#tutorial-1-learning-trajectories-with-monocle-3). Briefly, the top differentially expressed genes were selected as ‘ordering genes’ to recover lineage trajectories in Monocle3 using default parameters. After pseudotime was determined, differentially expressed genes were clustered to verify the fidelity of lineage trajectories.

### SCENIC analysis

SCENIC analysis was performed using utilizing pySCENIC (v 0.12.1) based on the hg38_refseq-r80_10kb_up_and_down_tss databases. pySCENIC was used to assess the enrichment of transcription factors and the activity of regulons in fibroblast subpopulations. First, TF-gene co-expression modules were reconstructed with GRNBoost2 in a data-driven manner. Subsequently, modules were trimmed by RcisTarget analysis and genes in the respective TF binding motifs were enriched. These significant gene regulatory networks are termed as regulons. Once the regulons were obtained, AUCell activity of each regulon across individual cell was evaluated and a binary regulon activity matrix was obtained.

### Cell culture

The HSF human fibroblast cell line was obtained from **American Type Culture Collection (ATCC**) and grown in DMEM medium (Gibco). All the media were supplemented with 10% fetal bovine serum (FBS, Gibco) and 1% antibiotics, and cells were grown in a humidified atmosphere with 5% CO2 at a temperature of 37 °C.

### Real-time quantitative PCR

Total RNA was extracted with TRIzol (Invitrogen), and TB Green-based real-time PCR was carried out with first-strand cDNA synthesis products generated from total RNA (TaKaRa, Japan). Relative mRNA expression was analyzed with the ∆∆Ct method **Methods 2001**,** 25**,** 402**. Sequences of the RT-qPCR primers of target genes are shown in **Table S2.**

### Transfection assay

The siRNAs of *Clock* gene in this article were purchased from RiboBio Corp. The antisense and sense siRNA sequences are shown here: Clock_SS: GGACAAGUCUACUGUUCUACA, Clock_AS: UAGAACAGUAGACUUGUCCAU. According to the manufacturer’s instructions, siRNAs were transfected into cells with Lipofectamine 3000 (Thermo Fisher Scientific, Inc.).

### Transwell assay

Transwell assays were carried out with 24-well Transwell plates (8-µm pore size; Millipore). 1 × 10^5^ cells transfected before were seeded on the upper chamber in serum-free medium, whereas the lower chamber contained medium with 20% FBS applied as a chemoattractant. After incubation for 24 h, the cells on the bottom surface of the filter were fixed with 4% paraformaldehyde, stained with hematoxylin/eosin dye, and counted.

### CCK8 and CSFE assay

The CCK8 assay was performed using a CCK8 kit (Beyotime) following the manufacturer’s protocol. Briefly, three type of HSF cells were plated into 96-well plates (5 × 10 3 cells per well) in 100 µl of culture medium or serum-free condition for 12, 24, 48 h and 72 h at 37 °C. CCK-8 solution (100 µl/well) was added for another 2 h and then incubated for 12, 24, 48, and 72 h. Then, the optical density (OD) was measured at 450 nm with a microplate reader (BioTek Synergy HT).

For the proliferation assay, different treated HSF cells were resuspended at 1 × 10 ^6^ cell/ml in PBS supplemented with 5% FBS and incubated with 5 µM CFSE (CellTrace CFSE Cell Proliferation Kit; Invitrogen) solution for 5 min at room temperature (RT). Stained cells were extensively washed and cultured for 3 days in complete DMEM medium supplemented or not with specific stimuli. Cell division was assessed by measuring the decrease in CFSE fluorescence via flow cytometry.

### Apoptosis assay

Cell apoptosis was assessed using Annexin V/propidium iodide double staining (BD Biosciences, CA, USA). HSF were seeded in 60-mm dishes (4 mL, 1 × 10^6^ /well) and allowed to incubate for 24 h. Subsequently, following siRNA_NC and siRNA_Clock with transfected into cells with Lip3000, after 3 days culture, the adherent cells were collected at specified time points and rinsed twice with ice-cold PBS. The cells were then suspended in binding buffer at a concentration of 1 × 10 ^6^ /mL and subjected to double staining with annexin V-FITC and propidium iodide, as per the manufacturer’s instructions. The resulting mixture was incubated in the dark for 15 min at room temperature and analyzed using the Beckman Coulter FC500 flow cytometry system and CXP software (Beckman Coulter, Fullerton, CA, USA). The apoptosis rate in this study encompasses both early and late apoptosis rates.

### TCGA data analysis

Data extraction and analysis from the Cancer Genome Atlas (TCGA) was performed using the online Gene Expression Profiling Interactive Analysis (GEPIA) GEPIA: http://gepia##cancer-pku##cn^45^. We used the Box Plot drawing option of the Expression DIY module of GEPIA. To analyze data, we selected the skin cutaneous melanoma (SKCM) datasets and used a Log2FC Cutoff value of 1 and a p -value cutoff of 0.01. The total number of tissues analyzed were: SKCM tumor, 461; adjacent normal tissue,558. To determined CLOCK and related other 10 gene expression, we furtherly using the TIMER2.0 website (http://timer##cistrome##org/ ). To analyze data, we selected the skin cutaneous melanoma (SKCM) datasets. The total number of tissues analyzed were: SKCM tumor, 103; SKCM Metastasis, 368.

### Statistical analysis

Data are expressed as the mean ± SD. Comparisons of cell growth were carried out with one-way analysis of variance (ANOVA) via SPSS 18.0. Differences between groups were determined by Student’s t test via Graph-Pad Prism 8.0. Differences for which P-values < 0.05 (two-sided) were considered statistically significant (*, *P* < 0.05; **, *P* < 0.01; and n.s, not significant).

## Supplementary Information

Below is the link to the electronic supplementary material.

Supplementary Material 1

Supplementary Material 2

## Data Availability

The data presented in the study are deposited in the GEO database and GSA repository, and the accession number are GSE163973, GSE160536 and PRJCA003143.
