# Supplementary material for: Single‑cell RNA sequencing reveals fibroblast heterogeneity and identifies CLOCK as a key regulator in fibrotic skin diseases
Source: Sci Rep. 2025 Dec 6;16:786. doi: 10.1038/s41598-025-30260-6 (PMC12779643; doi:10.1038/s41598-025-30260-6)
Supplement: Supplementary file 1 — Supplementary Material 1 [file 41598_2025_30260_MOESM1_ESM.docx]

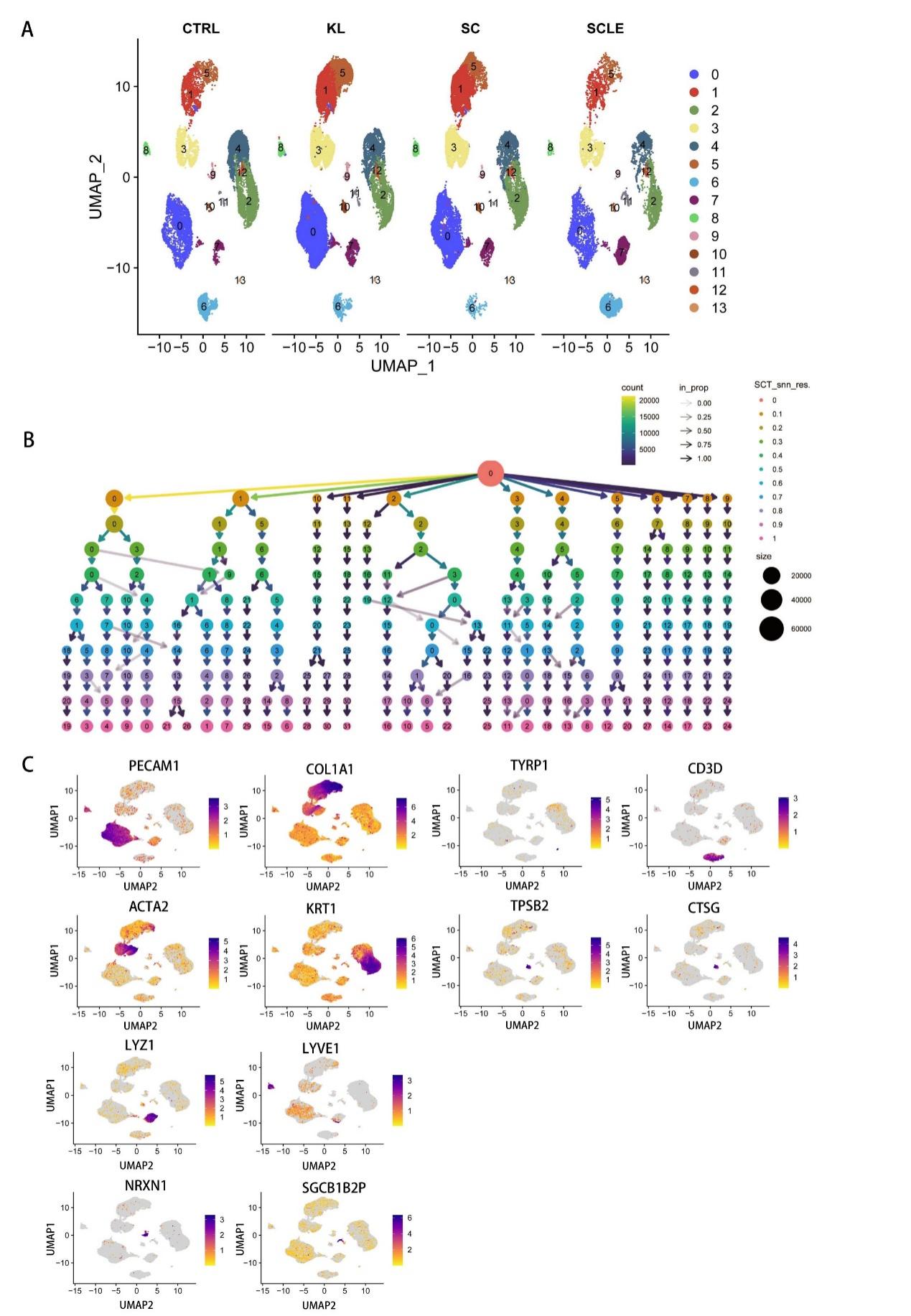


**SM.Fig.1** **(A)** The split UMAP of all cells in 4 different state including CTRL, KL, SC, and SCLE； **(B)** Clustering tree plot for the whole cells using various resolution parameters (from 0.1 to 1); **(C)** Feature plots of expression distribution for selected cluster-specific genes. Expression levels for each cell are color-coded and overlaid onto the UMAP plot.


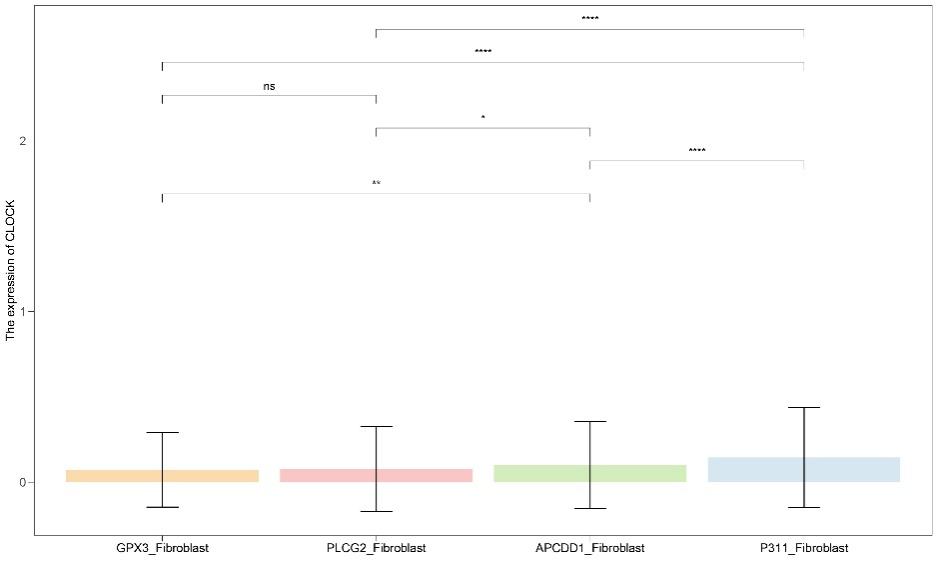


**SM. Fig. 2 Relative expression of CLOCK among 4 sub-fibroblast groups**

This figure illustrates the expression of the CLOCK gene in four distinct sub-fibroblast groups: GPX3_Fibroblast, PLCG2_Fibroblast, APCDD1_Fibroblast, and P311_Fibroblast. The box plots display differences in expression, with error bars representing the standard deviation. Statistical significance is indicated by asterisks: "ns" denotes non-significance, * indicates p < 0.05, ** indicates p < 0.01, *** indicates p < 0.001, and **** indicates p < 0.0001. Notably, CLOCK expression is significantly elevated in the P311_Fibroblast group compared to the others, suggesting its potential importance in fibroblast differentiation.


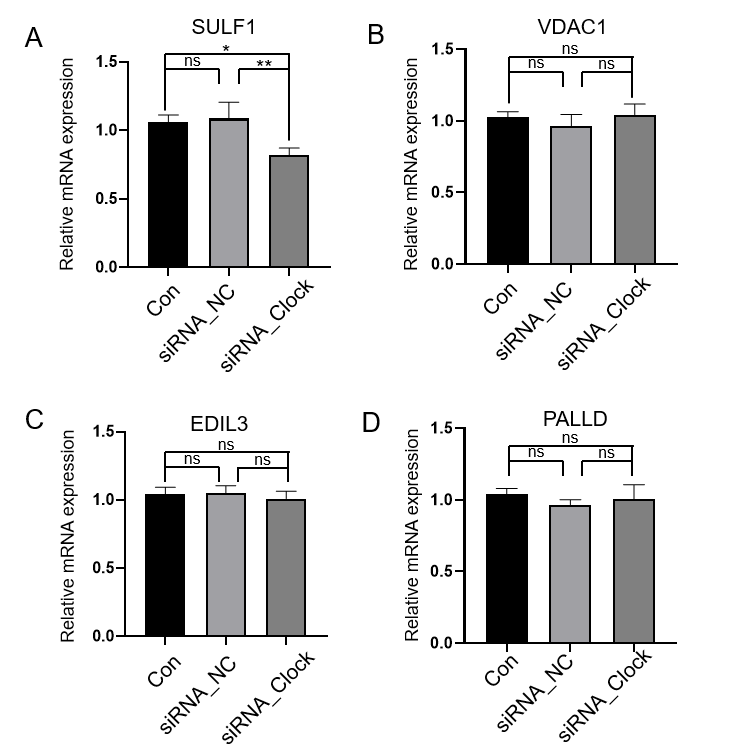


**SM. Fig. 3 The assessment of the four putative CLOCK targets (*EDIL3, PALLD, SULF1, and VDAC1*) in the siCLOCK-treated NIH-3T3 cell lines**

**(A-D)** The relative mRNA expression of *SULF1, VDAC1, EDIL3,* and *PALLD gene* with clock control siRNA (con), siRNA negative control (siRNA_NC), and siRNA_Clock (siRNA_clock with 10 nM).
