# Supplementary material for: Single‑cell RNA sequencing reveals fibroblast heterogeneity and identifies CLOCK as a key regulator in fibrotic skin diseases
Source: Sci Rep. 2025 Dec 6;16:786. doi: 10.1038/s41598-025-30260-6 (PMC12779643; doi:10.1038/s41598-025-30260-6)
Supplement: Supplementary file 2 — Supplementary Material 2 [file 41598_2025_30260_MOESM2_ESM.docx]

Table.S2 RT-primers sequences

| Gene name | primers |
| --- | --- |
| CLOCK-Forward | GCCTCAGCAGCAACAGCAGC |
| CLOCK -Reverse | ACCGCATGCCAACTGAGCGA |
